# SMPDL3B Predicts Poor Prognosis and Contributes to Development of Acute Myeloid Leukemia

**DOI:** 10.3389/fmolb.2021.695601

**Published:** 2021-08-24

**Authors:** Huiqing Qu, Ye Zhu

**Affiliations:** ^1^ Department of Blood Transfusion, Binzhou Medical University Hospital, Binzhou, China; ^2^ Department of Internal Medicine, People’s Fifth Hospital of Jinan City Affiliated to Weifang Medical University, Jinan, China

**Keywords:** acute myeloid leukemia, biomarker, SMPDL3B, prognosis, apoptosis

## Abstract

**Background:** Acute myeloid leukemia (AML), characterized by the low cure rate and high relapse, urgently needs novel diagnostic or prognostic biomarkers and potential therapeutic targets. Sphingomyelin Phosphodiesterase Acid Like 3B (SMPDL3B) is a negative regulator of Toll-like receptor signaling that plays important roles in the interface of membrane biology and innate immunity. However, the potential role of SMPDL3B in human cancer, especially in AML, is still unknown.

**Methods:** The expression of SMPDL3B in AML samples was investigated through data collected from Gene Expression Omnibus (GEO). Association between SMPDL3B expression and clinicopathologic characteristics was analyzed with the chi-square test. Survival curves were calculated by the Kaplan–Meier method. Cox univariate and multivariate analyses were used to detect risk factors for overall survival. The biological functions of SMPDL3B in human AML were investigated both *in vitro* and *in vivo*.

**Results:** Expression of SMPDL3B mRNA was significantly upregulated in human AML samples and closely correlated to cytogenetics risk and karyotypes. Elevated expression of SMPDL3B was associated with poor overall survival and emerged as an independent predictor for poor overall survival in human AML. Blocked SMPDL3B expression inhibited AML cells growth both *in vitro* and *in vivo* via promoting cell apoptosis.

**Conclusion:** Taken together, our results demonstrate that SMPDL3B could be used as an efficient prognostic biomarker and represent a potential therapeutic target for human AML.

## Introduction

Acute myeloid leukemia (AML), characterized by the uncontrolled proliferation and accumulation of granulocyte or monocyte precursors in the bone marrow and peripheral blood, is the most common type of leukemia in adults (El Omri et al., 2020). It is estimated that there are 21,450 new cases of AML in the United States in 2019, with an annual incidence of 4.2 per 100,000 persons (Lai et al., 2019). Although new therapeutic approaches have improved outcomes in the treatment of AML in the last decades, AML has the lowest survival rate of all leukemia due to the high rate of relapse. The 5-year overall survival of patients with AML is still unsatisfactory (only 38% for younger patients, and <10% for older patients) (Kantarjian et al., 2021). Thus, the effective treatment of AML and novel personalized therapies for AML patients are urgently needed.

An increasing number of studies have revealed that AML displays a complex variety of genetic changes, which results in the malignant proliferation of AML cells and variable clinical prognosis of AML patients (Deng et al., 2018; Lai et al., 2019; Ma et al., 2019; Martin et al., 2019). These mutation and abnormal expression genes associated with AML provide significant prognostic information for determining the response to chemotherapy and survival outcome (DiNardo et al., 2021; Xuan et al., 2020; Yang et al., 2020). Consequently, a better understanding of these changes is essential for the effective treatment of AML and the design of novel personalized therapies.

The SMPDL3B (Sphingomyelin Phosphodiesterase Acid Like 3B) protein, a phosphodiesterase, plays important roles in cell membrane lipid-modulation and membrane fluidity (Mitrofanova et al., 2019; Yoo et al., 2015). SMPDL3B acts as a negative regulator of Toll-like receptor signaling and changes the cellular lipid composition and membrane fluidity in macrophages (Heinz et al., 2015). Moreover, excess SMPDL3B was reported to impair insulin receptor isoform B-dependent signaling by interfering with insulin receptor isoforms binding to caveolin-1 in podocytes in diabetic kidney disease (Mitrofanova et al., 2019). In addition, SMPDL3B modulated radiation-induced damage of human glomerular endothelial cells (Abou Daher et al., 2020) and renal podocytes (Ahmad et al., 2017). Recently, Frank W. and colleagues suggested that the elevated expression of SMPDL3B significantly correlated with poor survival of prostate cancer patients (Waldbillig et al., 2020). Moreover, the knockdown of SMPDL3B impaired the migration of PC3 cells (Waldbillig et al., 2020). However, the clinical significance and biological function of SMPDL3B in human AML have not been explored. In the present study, we aimed to investigate the expression of SMPDL3B in AML patients. Moreover, the association of SMPDL3B expression with clinical outcomes of AML patients was also explored. Furthermore, the roles of SMPDL3B in supporting AML cells growth were investigated both *in vitro* and *in vivo*.

## Materials and Methods

### Cell Culture and Cell Growth/Apoptosis Assays

The human AML cell lines Kasumi-1, NB4, HL-60, THP-1, U937, MV4-11, and HEL cell lines were purchased from the Cell Bank of the Shanghai Institute for Biological Sciences (Chinese Academy of Sciences, Shanghai, China). The leukemia cell lines were grown in RPMI-1640 and HEK-293T cells were grown in high glucose DMEM medium supplemented with 10% fetal bovine serum. All the cells were maintained at cell culture incubator at 37°C and 5% CO_2_. For the cell growth assays, the sorted GFP + AML cells (infected with Scramble or SMPDL3B shRNA lentivirus) were seeded in 24-well plate at the indicated numbers. The cell growth was evaluated by calculating the living cell number with TC20™ automated cell counter (Bio-Rad, Hercules, CA) by trypan blue dyeing at the indicated days. For the apoptosis assay, GFP + AML cells (infected with sgRNA lentivirus) were stained by Annexin V-PE/7-AAD apoptosis detection kit (#A213-01, Vazyme) as the instructions. At least 10,000 cells were collected by FACS to determine the percentage of the apoptotic cells. All the experiments were repeated three times.

### *In Silico* Data Collection

The gene expression profile of SMPDL3B included 2096 blood or bone marrow samples of acute and chronic leukemia patients based on the platform of Affymetrix HG-U133 Plus 2.0 GeneChips was downloaded from the GEO (Gene Expression Omnibus) database (GSE13159) (https://www.ncbi.nlm.nih.gov/geo/query/acc.cgi?acc=GSE13159). The cBio Cancer Genomics Portal (c-BioPortal) was used to download SMPDL3B mRNA expression and clinicopathological data in 200 AML patients (http://cbioportal.org).

### GO and KEGG Pathway Enrichment Analysis

Candidate genes correlated with SMPDL3B in human AML patients were downloaded from the Gene Expression Profiling Interactive Analysis (GEPIA) website (http://gepia.cancer-pku.cn/). A total of 66 genes with the │Pearson correlation coefficient│> 0.6, *p* < 0.05, were included and listed in Sup. Table 1. The gene ontology resource (http://geneontology.org/) was employed to perform GO (gene ontology) functional annotation and KEGG (Kyoto Encyclopedia of Genes and Genomes) pathway enrichment analysis for these SMPDL3B correlated genes. The GO annotation analysis contained three categories, including cellular component (CC), biological process (BP), and molecular function (MF).

**TABLE 1:** Association between SMPDL3B expression and clinicopathological characteristics in AML patients.

Patient’s parameters	SMPDL3B ^low^, 64	SMPDL3B ^high^, 64	*p*
Sex, male/female	35/29	33/31	0.7232
Age, <60/≥60	33/31	38/26	0.3739
WBC (median, range) ×10^9^/L	29 (0.4–224)	12.5 (1–203)	0.4222
Hemoglobin (median, range) g/dL	9 (6–13)	10 (7–13)	0.7582
Platelets (median, range) ×10^9^/L	47.5 (9–174)	42.5 (9–232)	0.2833
BM blasts (median range)%	22 (1–97)	45 (1–97)	0.0627
FAB classifications			0.0549
M0	5	8	
M1	11	18	
M2	14	17	
M3	6	9	
M4	17	6	
M5	11	4	
M6	0	1	
M7	0	1	
Cytogenetics risk			0.0014*
Favorable	15	14	
Intermediate	42	26	
Poor	7	24	
Karyotypes			<0.0001
Normal	40	25	
inv (16)	8	0	
t (8; 21)	1	5	
t (15; 17)	6	7	
Complex	2	16	
Others	7	11	
Gene mutations			
FLT3 mutation, P/N	19/45	18/46	0.8454
Activated RAS, P/N	5/59	3/61	0.4652
NPM1, P/N	17/47	13/51	0.4039
IDH1, P/N	11/53	12/52	0.8179

**TABLE 2:** Univariate and multivariate Cox regression analysis of overall survival in AML patients.

Characteristics	HR	Univariate 95% CI	*p*	HR	Multivariate 95% CI	*P*
Sex (female vs. male)	1.047	0.6751–1.626	0.8373			
Age (≥60 vs. < 60)	2.719	1.979–5.061	**< 0.0001**	1.883	1.130–3.140	**0.0152**
WBC (≥median vs. < median)	1.206	0.7756–1.913	0.3990			
Hemog (≥median vs. < median)	1.350	0.8809–2.145	0.1694			
Platelets (≥median vs. < median)	1.425	0.9245–2.222	0.1116			
BM blasts (≥median vs. < median)	1.262	0.8178–1.982	0.2912			
FAB classifications	///	///	**0.0146**	1.265	0.784–1.372	0.463
(M0 vs. M3)	3.562	1.334–12.43	**0.0183**			
(M1 vs. M3)	3.624	1.370–7.132	**0.0092**			
(M2 vs. M3)	2.787	1.037–5.792	**0.0467**			
(M4 vs. M3)	4.287	1.679–8.939	**0.0025**			
(M5-7 vs. M3)	3.643	1.371–9.821	**0.0127**			
Cytogenetics risk	///	///	**0.0001**	1.559	1.086–2.237	**0.0161**
(Poor vs. favorable)	3.313	1.764–5.754	**0.0002**			
(Intermediate vs. favorable)	2.481	1.249–4.050	**0.0077**			
Karyotypes			0.0662			
FLT3 (mutation vs. normal)	0.8857	0.5549–1.413	0.6145			
NPM1 (positive vs. negative)	1.121	0.6755–1.882	0.6493			
IDH1 (positive vs. negative)	2.423	1.739–8.236	**0.0011**	1.821	1.016–3.257	**0.0439**
RAS (positive vs. negative)	0.6608	0.3022–1.616	0.4091			
SMPDL3B (high vs. low)	2.139	1.367–3.287	**0.0009**	1.927	1.186–3.125	**0.0079**

### RNA Extraction and Quantitative Real-Time PCR (qRT-PCR) Analysis

Cells were lysed and the total RNAs were extracted by using TRIzol Reagent (Invitrogen, Carlsbad, CA). cDNAs were reverse transcribed with the iScript^TM^ cDNA Synthesis Kit (Bio-Rad). SMPDL3B and reference GAPDH were amplified by qRT-PCRs performed in the QuantStudio 5 Real-Time PCR machine using the iTaq Universal SYBR Green Supermix (Bio-Rad). Relative SMPDL3B mRNA expression levels were calculated by using the ΔΔCt method, normalized to GAPDH. The following PCR primers were used: SMPDL3B forward: 5′-TGG​TCA​ATG​GGG​CCA​ACA​AT -3′ SMPDL3B reverse: 5′-GGT​GGA​AGG​AGC​TCA​ACC​TT-3′ GAPDH forward: 5′-GAATGGG CAGCCGTTAGGAA-3′, GAPDH reverse: 5′-AAA​AGC​ATC​ACC​CGG​AGG​AG-3’. All the primers used in this study were synthesized by Sangon Biotech (Shanghai, China).

### Western Blot Assay

The cells were collected and lysed with RIPA lysate buffer containing 1 mmol/L PMSF (#ST505, Beyotime) and 0.1 g/L leupeptin (#SG 2012, Beyotime, Shanghai). Protein samples were quantified with BCA. For the Western blotting, 30 µg of total protein was loaded into SDS-PAGE gel and transferred to the nitrocellulose membrane. The membrane then was blocked and incubated with SMPDL3B antibody (#16552-1-AP, Proteintech) and HRP-conjugated secondary antibody. The housekeeping gene GAPDH was used as an internal control.

### CRISPR–Cas9-Mediated Gene Knockout in AML Cells

SMPDL3B-knockout (SMPDL3B-KO) cell lines were performed as described previously (Yamauchi et al., 2018). Briefly, AML cells were infected with pCW-Cas9 (#50661, Addgene, Watertown, MA, United States) lentivirus and selected with 1 μg/ml puromycin treatment. Then, the cells were infected with the sgRNA lentivirus. Scramble control sgRNA (sgRNA: 5′-CCA​CAC​CTG​TCT​AGC​ATG​AC-3′) or SMPDL3B targeting sgRNA (sgRNA1: 5′-ATG​GAC​TCA​TTA​CTA​AGC​CA-3′; sgRNA2: 5′- ATG​GAC​TCA​TTA​CTA​AGC​CA -3′) were cloned into the sgRNA plasmid pLenti-sgRNA (#89638, Addgene), individually. At 7 days after treatment with 1 μg/ml doxycycline, GFP + cells were seeded into a 96-well plate as a single cell per well. After cell expansion, knockout clones were verified by Western blotting.

### Xenograft Tumor Model Assay

The animal assays were performed according to the guidelines and approval of the Ethical Committee of Binzhou Medical University. To establish xenograft models, 5 × 10^6^ SMPDL3B-WT (left) or SMPDL3B-KO (right) THP-1 cells were subcutaneously injected into the flanks of 4-week-old female athymic BALB/c nude mice (Vital River, Beijing). At 10 days after injection, the size of the xenografted tumor was measured every 3 days by using a vernier caliper. At 25 days after injection, the mice were sacrificed, and the xenograft tumors were stripped. The xenograft tissues were subjected to TdT-mediated dUTP Nick-End Labeling (TUNEL) analysis.

### TUNEL Assay

In order to assess the apoptosis of the xenograft tumors, the slices of the tumor tissue were baked and rehydrated, then antigen repair was performed. The DNA fragmentation was determined by the TdT-mediated dUTP nick end-labeling (TUNEL) Kit (#C1086, Beyotime, Shanghai, China.) according to the manufacturer’s instructions. Briefly, the slices were incubated in H_2_O_2_ solution and then washed by PBS. Next, the slices were incubated with immunostaining washing solution and TUNEL staining solution. The fluorescent images were observed under a fluorescence microscope (Nikon Corp., Tokyo, Japan). The results were analyzed by IMAGE J software.

### Statistical Analysis

Statistical analysis was performed using SPSS 24.0 statistical software (SPSS Inc., Chicago, IL, United States). Data with normal distribution were expressed as mean ± S.D. *t*-test (2 groups), one-way ANOVA test (3 or more groups), Pearson’s chi-square test, Spearman’s correlation analysis, and Fisher’s exact test were used to compare variables. The Kaplan–Meier method, log-rank test, and Cox’s proportional hazards model were used for survival analysis.

## Results

### SMPDL3B Is Highly Expressed in AML Patients and Correlates With Clinical Characteristics

**FIGURE 1:**
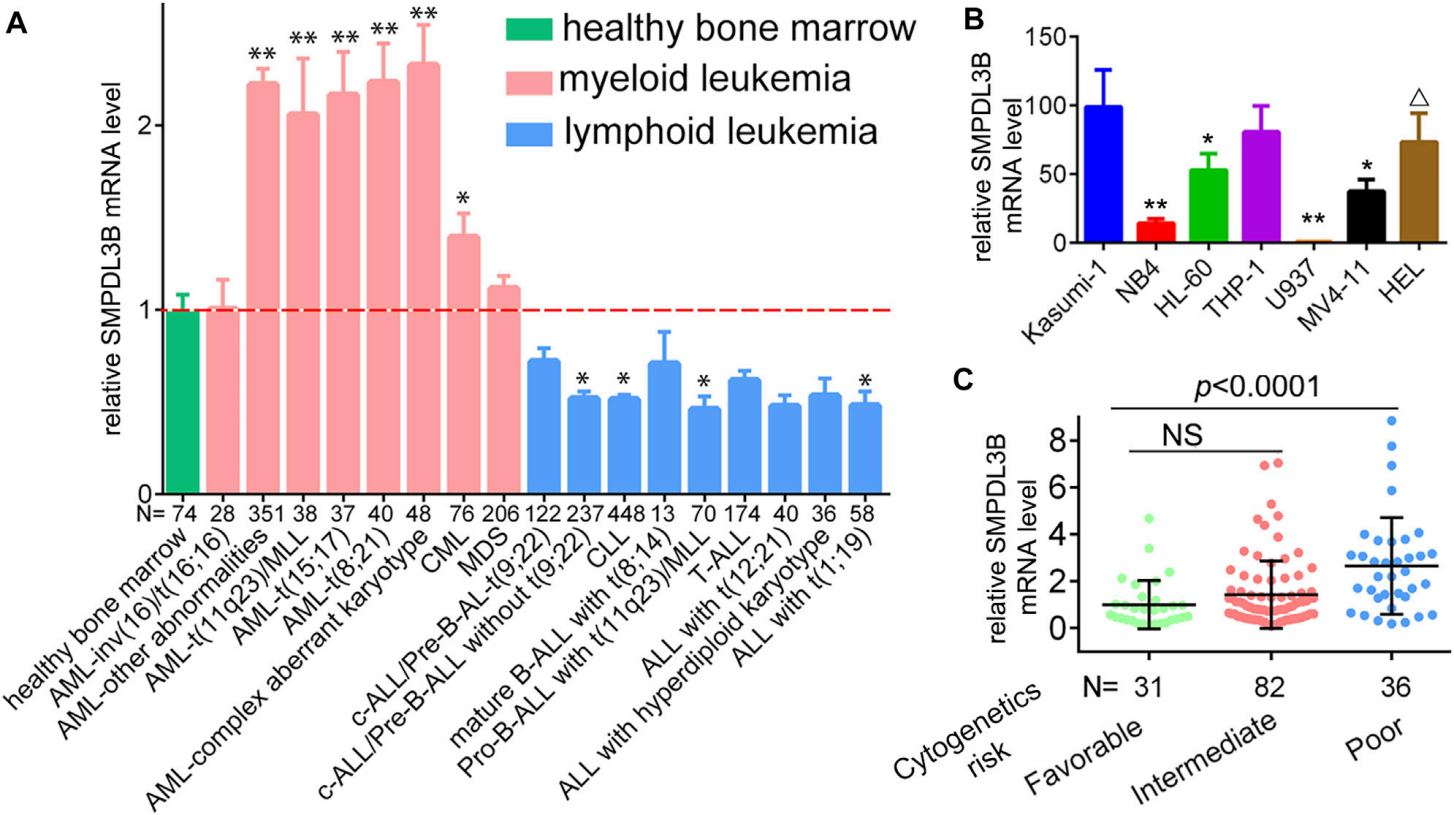
SMPDL3B is highly expressed in AML patients and cell lines. **(A)** Relative mRNA expression of SMPDL3B in different karyotypes of AML vs. healthy samples. *p < 0.005, **p < 0.001 compared to healthy control. (Data were obtained from GSE13159.) **(B)** qRT-PCR analysis of SMPDL3B mRNA expression in the indicated cell lines. All the levels of SMPDL3B mRNA expression were normalized by GAPDH. **(C)** Relative SMPDL3B mRNA expression in human AML patients with indicated cytogenetics risk. The data were obtained from the TCGA AML database.

To evaluate whether the expression of SMPDL3B is connected to AML evolution and advancement, we initially examined its expression by *in silico* analysis. Herein, the microarray data derived from the GEO database (GSE13159) were analyzed. SMPDL3B was expressed at significantly higher levels in almost all types of myeloid leukemia cells tested as compared to healthy bone marrow samples, whereas SMPDL3B was expressed at lower levels in lymphoid leukemia cells (Figure 1A). Consistently, the qRT-PCR analysis showed that SMPDL3B mRNA was highly expressed in several human AML cell lines, including Kasumi-1, THP-1, HL-60, MV4-11, and so forth, but not in U937 cells (Figure 1B). Furthermore, the SMPDL3B mRNA expression was tested in a cohort of AML patients according to its cytogenetics risk. The results indicated that the expression of SMPDL3B mRNA was significantly higher in the patients with poor cytogenetics, whereas it was relatively lower in the patients with good or intermediate cytogenetics (Figure 1C). Together, these results indicated that SMPDL3B mRNA was remarkably increased in AML cells. To further explore the correlation between SMPDL3B mRNA expression and clinical characteristics in human AML, patients were divided into two groups based on the expression level of SMPDL3B (low: below the 50th percentile; high: above the 50th percentile). Statistical analysis revealed that expression of SMPDL3B was closely correlated to cytogenetics risk (*p* = 0.0014) and karyotypes (*p* < 0.0001), and it was a trend toward FAB classifications (*p* = 0.0549). However, SMPDL3B mRNA expression was not related to other clinical characteristics (*p* > 0.05) (Table 1).

### High Expression of SMPDL3B Predicts Poor Survival in Human AML Patients

**FIGURE 2:**
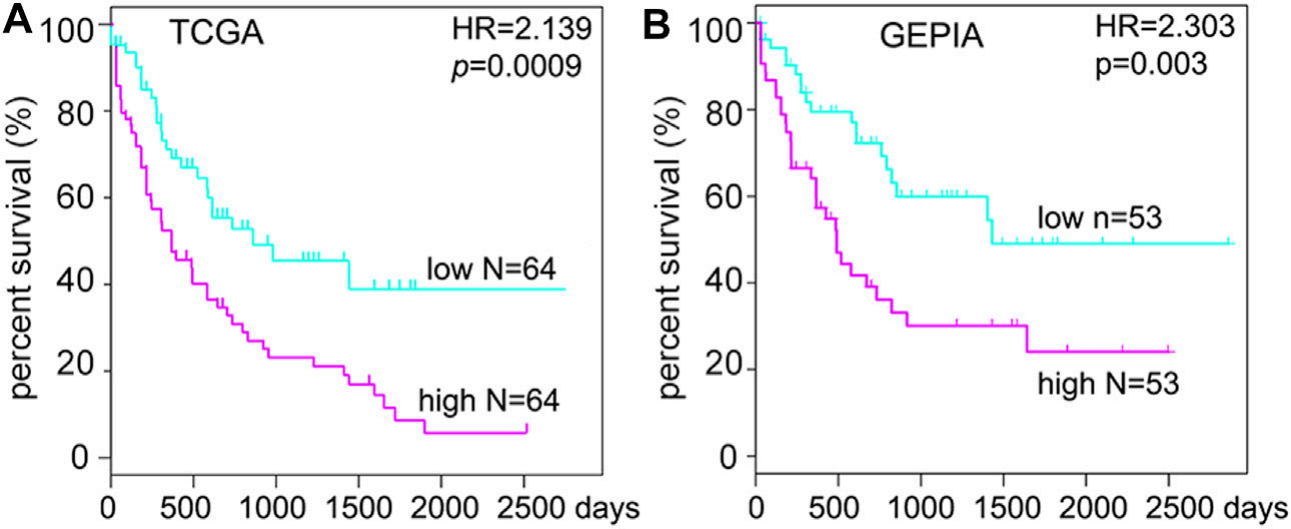
SMPDL3B mRNA expression levels negatively correlate with the overall survival of human AML patients. Kaplan–Meier analysis of overall survival in AML patients relative to SMPDL3B mRNA expression levels. (High: above the 50th percentile; low: below the 50th percentile.) Data were obtained from the TCGA AML database **(A)** and GEPIA **(B),** respectively. *p* value was calculated by the log-rank test.

**FIGURE 3:**
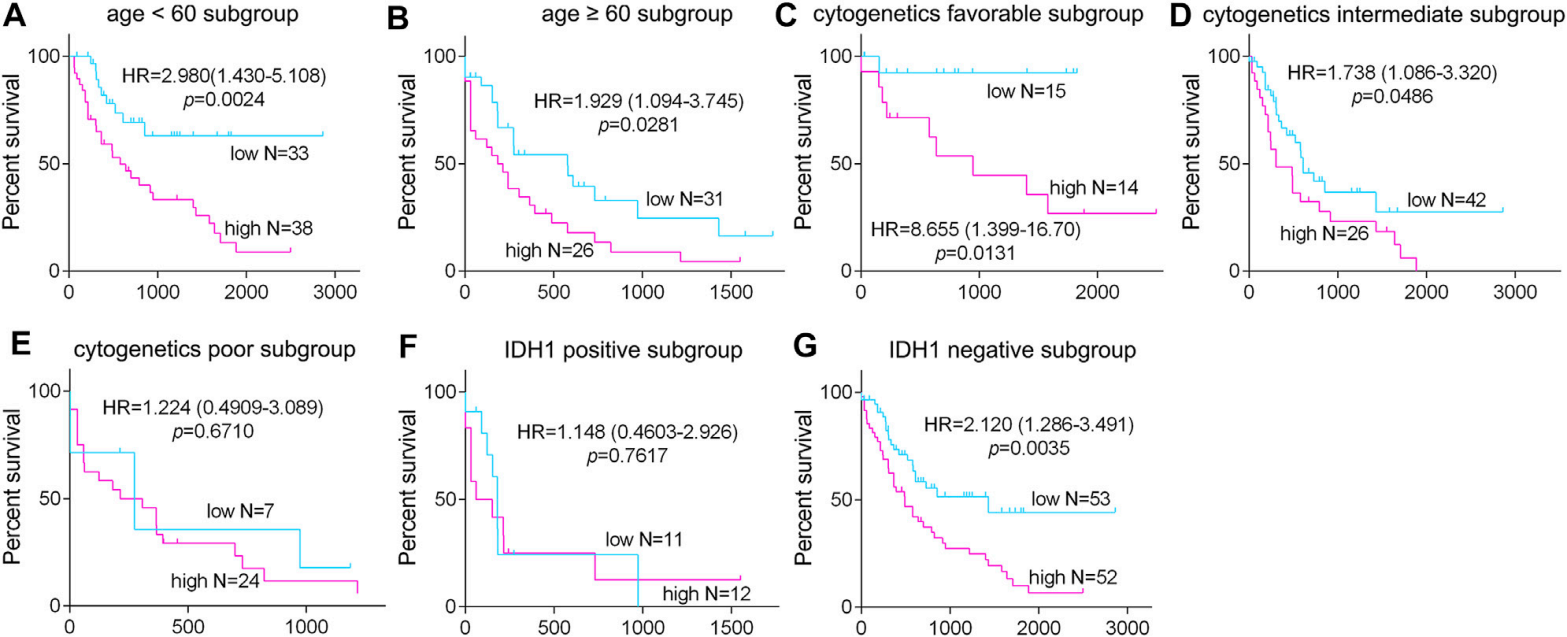
The expression of SMPDL3B in AML subtypes and correlation with overall survival of the patients. The AML patients were divided into the indicated subgroups (**(A)**: age <60, **(B)**: age ≥60; **(C)**: cytogenetic favorable, **(D)**: cytogenetic intermediate, **(E)**: cytogenetic poor; **(F)**: IDH1 positive, and **(G)**: IDH1 negative) according to the age, cytogenetics risk, and IDH1 expression status, respectively. The Kaplan–Meier overall survival in the indicated subgroups of AML patients with SMPDL3B low and SMPDL3B high expression is plotted respectively. *p* value was calculated by log-rank test.

To further detect the role of SMPDL3B in AML propagation, the overall survival rate was performed by the Kaplan–Meier analysis based on SMPDL3B expression. The Kaplan–Meier analysis revealed that the AML patients with high levels of SMPDL3B mRNA had a significantly poorer overall survival in the TCGA and GEPIA groups, respectively (Figures 2A,B). To test whether this finding was independent from the well-established prognostic indicators, such as age, FAB classification, cytogenetics risk, and karyotypes. Cox regression analyses (univariate and multivariate) of each of the clinicopathological variables with SMPDL3B mRNA expression were conducted. Univariate analysis revealed that the overall survival of AML patients significantly correlated with age, FAB classifications, cytogenetics risk, IDH1, and SMPDL3B expression level (all *p* < 0.05). Further, multivariate analysis was used to analyze all the statistically significant variables revealed by univariate analysis. SMPDL3B mRNA expression level (HR = 1.927, *p* = 0.0079), together with age, cytogenetics risk, and IDH1, was a significant independent prognostic factor for overall survival of AML patients. Consistent with these results, the overall survival rates were significantly different between SMPDL3B low and SMPDL3B high AML patients in preplanned age <60, age ≥60, cytogenetics risk favorable, cytogenetics intermediate, and IDH1 negative subgroups (Figures 3A–D,G). However, there was no difference of the overall survival in cytogenetics poor and IDH1 positive subgroups; such paradox might be due to the smaller sample size (Figures 3E,F). Together, these results suggested that high SMPDL3B mRNA expression served as an independent poor prognostic biomarker associated with decreased overall survival in human AML patients.

### Downregulation of SMPDL3B Inhibited Growth of Leukemia Cells Both *In Vitro* and *In Vivo*

**FIGURE 4:**
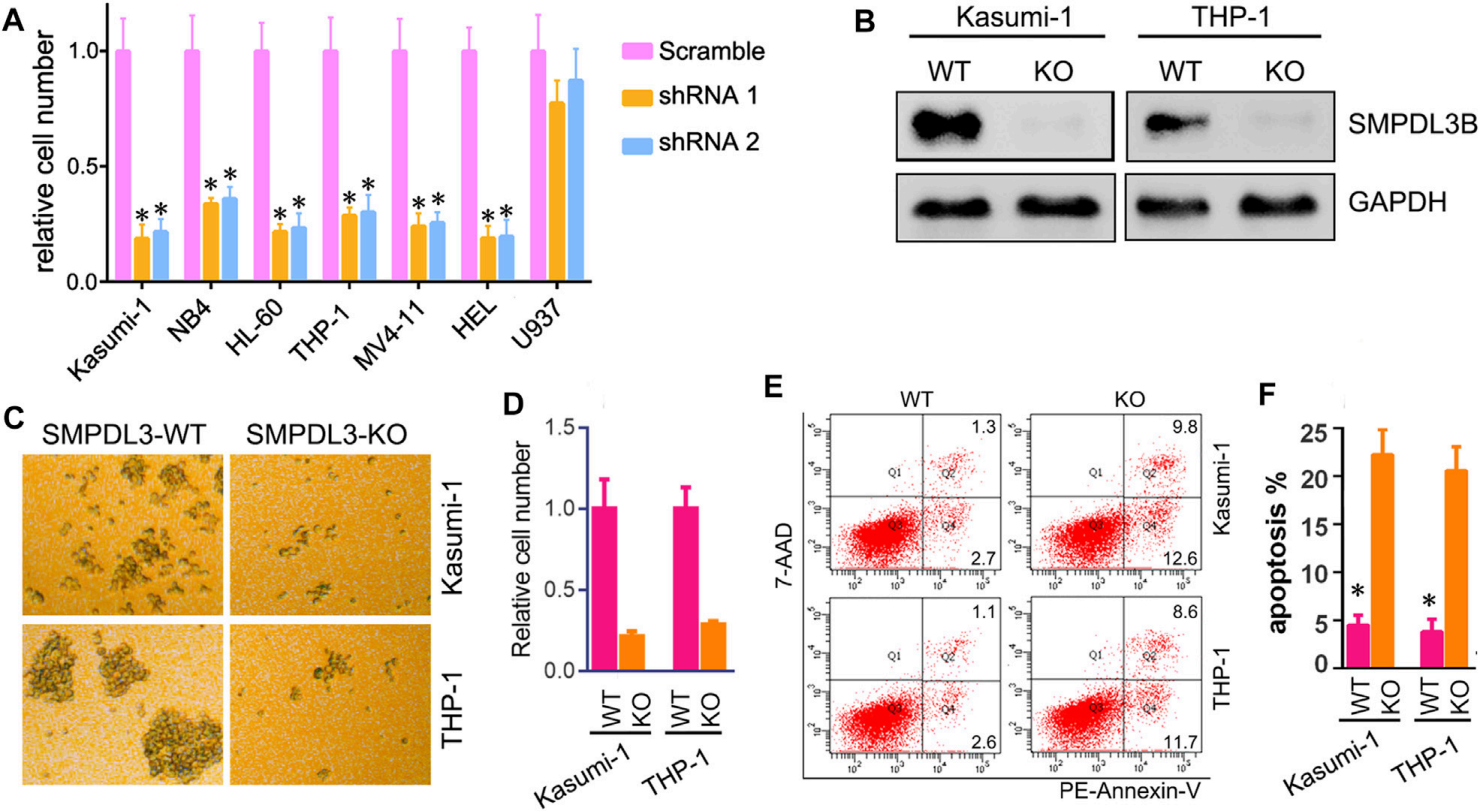
Inhibition of SMPDL3B suppressed growth and promoted apoptosis of AML cells. **(A)** The indicated AML cells were infected with Scramble or SMPDL3B shRNA lentivirus. The cell numbers were counted at 4 days after infection. The relative cell number is plotted. *n* = 5, *p < 0.001 compared to the Scramble group. **(B)** Western blot analysis of the expression of SMPDL3B in CRISPR/Cas9-edited AML cells. **(C)** Representative images of Kasumi-1 and THP-1 cells with wild type (WT) or knockout (KO) SMPDL3B. **(D)** The indicated SMPDL3B-WT or KO cells were seeded in 24-well plate. Cell numbers were counted at 4 days after being seeded. The relative cell number is plotted. *n* = 5, *p* < 0.001 compared to WT group. **(E)** Representative scatter plots showing apoptosis in SMPDL3B-WT and SMPDL3B-KO AML cells by annexin V-PE and 7-AAD staining and flow cytometry. **(F)** Quantification of the apoptotic cells. *n* = 3, *p < 0.001 SMPDL3B-WT vs. SMPDL3B-KO.

To further study the biological function of SMPDL3B in AML cells, we knocked down the expression of SMPDL3B via lentivirus-encoded shRNAs. The qRT-PCR results showed that the shRNA could efficiently decrease the expression of SMPDL3B (data not shown). Remarkably, the CCK-8 results indicated that knockdown of SMPDL3B expression could decrease the growth of each of those leukemia cell lines that express SMPDL3B (Figure 4A). In contrast, the tested shRNA did not influence the growth of U937 cells (Figure 4A). Since Kasumi-1 and THP-1 cells had the highest expression level of SMPDL3B of the cultured cells evaluated, we constructed SMPDL3B knockout Kasumi-1 and THP-1 leukemia cell lines via the CRISPR/Cas9 system and used these two cell lines in subsequent experiments (Figure 4B). As expected, knockout of SMPDL3B induced visible cell growth inhibition both in Kasumi-1 and THP-1 cells (Figures 4C,D). Notably, the knockout of SMPDL3B significantly increased the rate of apoptosis of AML cells (Figures 4E,F).

**FIGURE 5:**
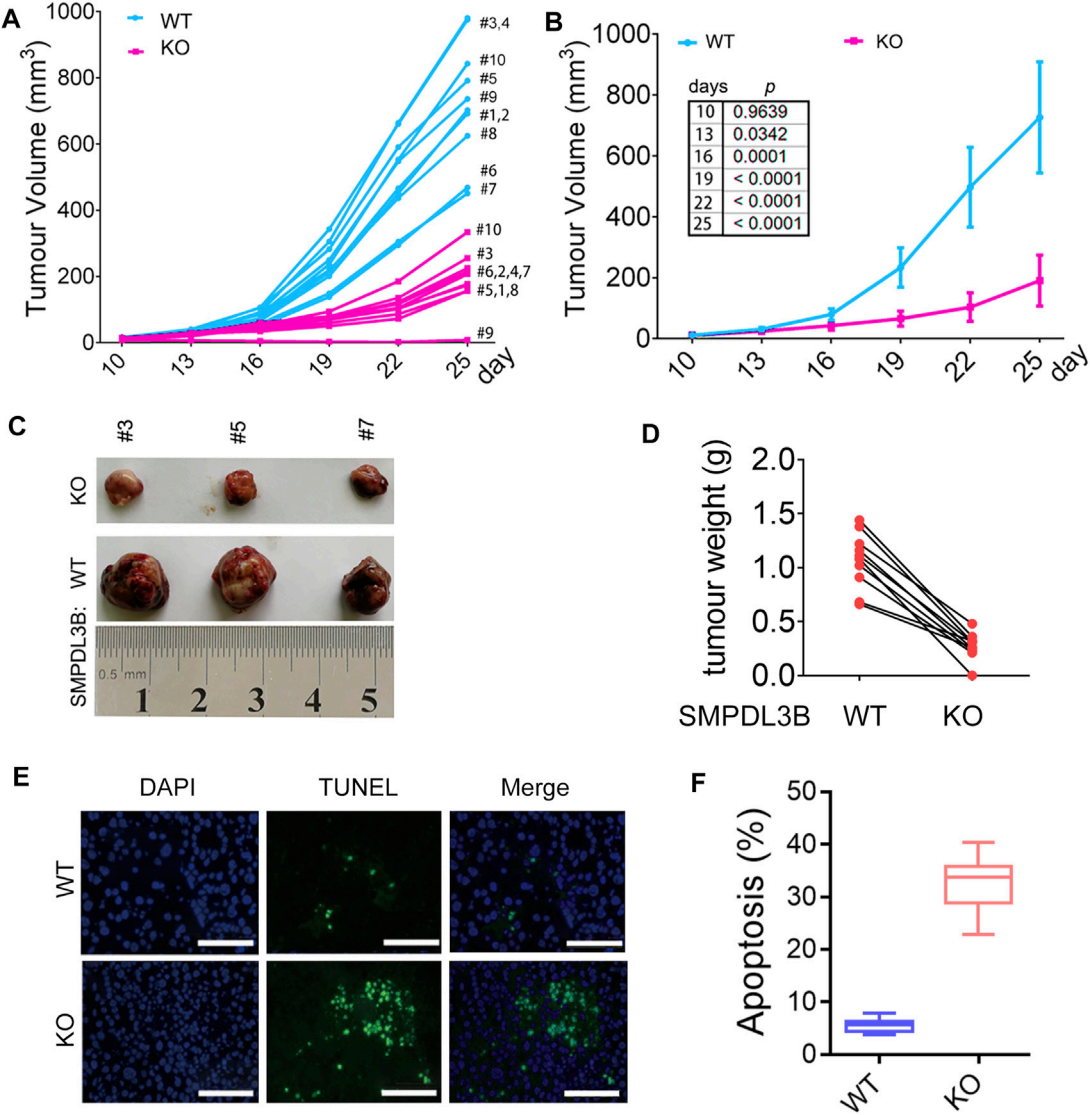
Knockdown of SMPDL3B expression suppressed the growth of AML xenograft tumors *in vivo*. The nude mice received a subcutaneous injection of SMPDL3B-WT or SMPDL3B-KO Kasumi-1 cells were sacrificed on day 25 after injection. **(A)** The tumor volume of each xenograft at the indicated days after injection was shown. **(B)** The average volume of the tumor xenografts was shown. Data were presented as mean ± S.D. (*n* = 10). **(C)** Representative images of the isolated tumors. **(D)** Each paired weight of the tumor xenografts was shown (*n* = 10, paired *t*-test, *p* < 0.001). **(E)** Representative images of the TUNEL assays for the tumors were shown (bars, 100 μM). **(F)** Percent apoptosis of the SMPDL3B-WT and KO tumors. *n* = 5, mean ± S.D. *p* < 0.01.

To further confirm whether SMPDL3B knockout had suppressive effects on AML tumor growth *in vivo*, the Kasumi-1 cells with wild type SMPDL3B (SMDPL3B-WT) or knockout SMPDL3B (SMPDL3B-KO) were subcutaneously injected into nude mice for xenograft. Herein, each of the xenograft tumors grown from the SMPDL3B-KO cells had a smaller volume than that grown from SMPDL3B-WT cells (Figures 5A,B). On day 25 after injection, the mean tumor volume was 726.6 mm^3^ in the WT group compared with 190.88 mm^3^ in the KO group, with the inhibitory rate being 73.73% (Figure 5B). Furthermore, all tumors were removed, photographed (Figure 5C), and weighed (Figure 5D) on day 25 after injection. The average tumor weights in WT and KO groups were 1.066 g and 0.3033 g, respectively. Statistical analysis showed significant suppression from the SMPDL3B-KO (Figure 5D). Moreover, the DNA fragmentation assay in tumor tissue showed that the SMPDL3B-KO tumor had a visible increase in cell apoptosis compared to the SMPDL3B-WT tumor (Figures 5E,F). Together, our results indicate that SMPDL3B contributed to AML cells growth both *in vitro* and *in vivo* and might be via inhibiting cell apoptosis.

### GO and KEGG Analyses for SMPDL3B Related Genes

**FIGURE 6:**
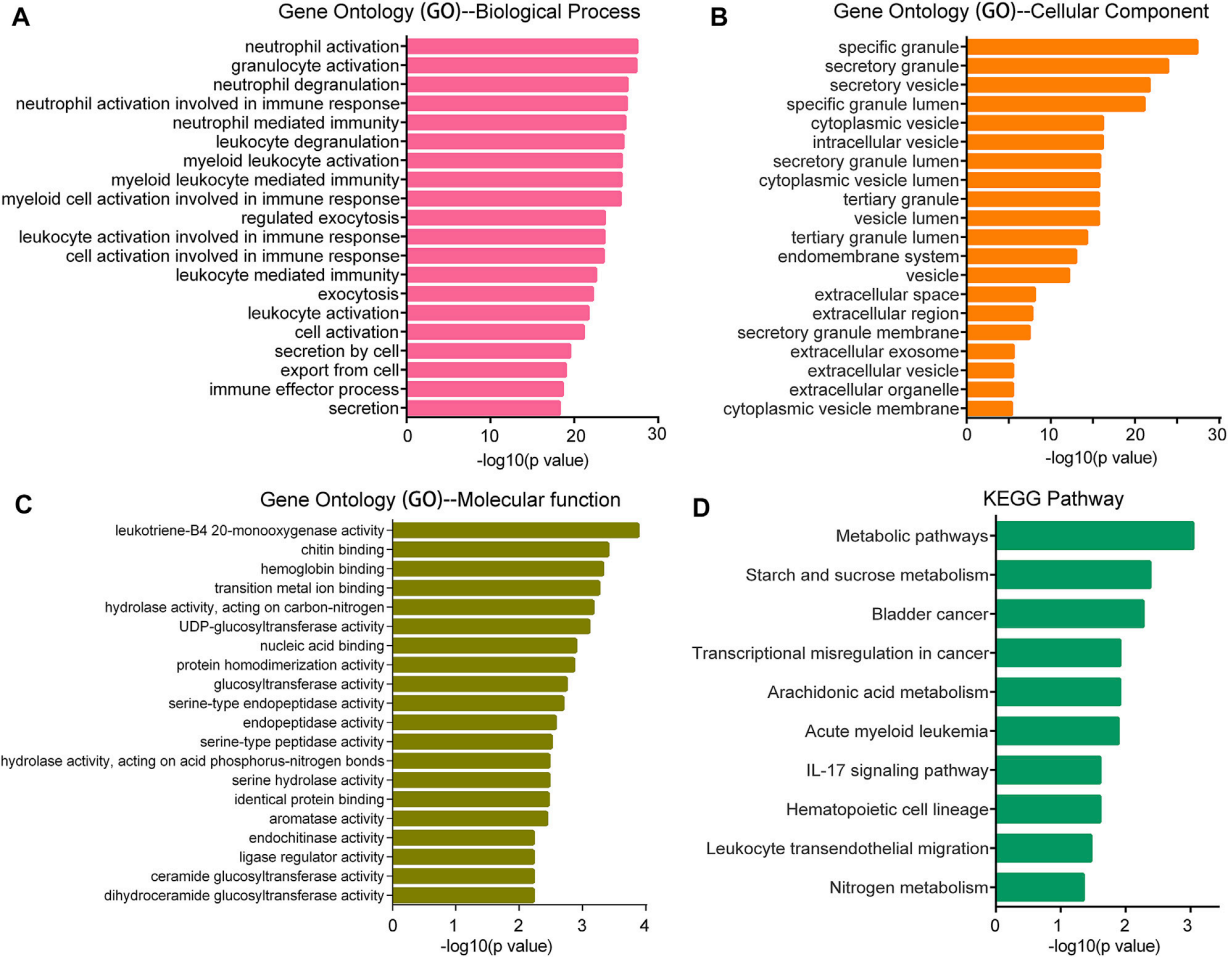
GO and KEGG pathway enrichment analysis for the SMPDL3B correlated genes. The top 20 most enriched GO biological process **(A)**, GO cellular component **(B)**, GO molecular function **(C)** categories, and the top 10 most enriched KEGG pathways **(D)** are plotted.

**FIGURE 7:**
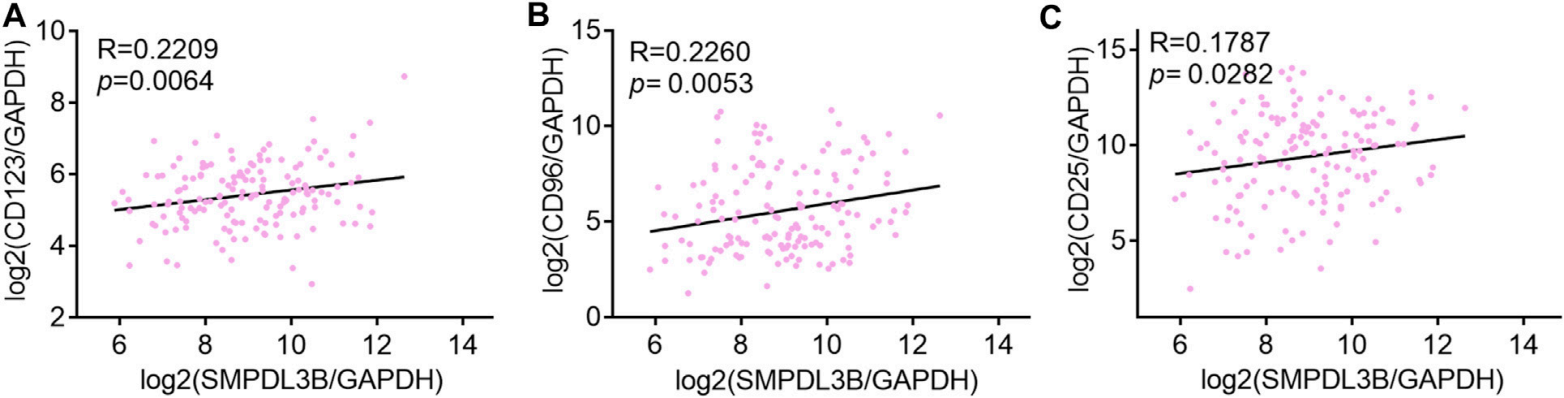
SMPDL3B expression positively correlates with CD123, CD96, and CD25 AML patients. **(A–C)** The log2 transcription level of SMPDL3B, CD123 **(A)**, CD96 **(B)**, and CD25 **(C)** in AML patients was shown after normalized to GAPDH. *n* = 151, Spearman analysis *p* < 0.05.

For further interpretation of the mechanism of SMPDL3B contributing to human AML cells growth, the gene expression profile datasets were downloaded from the GEO database (GSE13159). The Sanger Box analysis tool was applied to detect the SMPDL3B correlated genes, using adjust *p* value <0.05 and |R| ≥ 0.6 as cut-off criteria. After integrated bioinformatical analysis, a total of 66 genes were identified from the database (Supplementary Table S1). Furthermore, the gene ontology (GO) functional annotation was performed using the gene ontology resource online. GO analysis results showed that SMPDL3B correlated genes were particularly enriched in biological processes (BP), including neutrophil activation, granulocyte activation, neutrophil degranulation, and neutrophil mediated immunity (Figure 6A). For GO cell component (CC), the SMPDL3B correlated genes also were enriched in specific granule, secretory granule, secretory vesicle, and specific granule lumen (Figure 6B). In addition, molecular function (MF) analysis displayed that the SMPDL3B correlated genes were significantly enriched in leukotriene-B4 20-monooxygenase activity, chitin binding, hemoglobin binding, and transition metal ion binding (Figure 6C). In addition, we found that the most significantly enriched KEGG pathways of the SMPDL3B related genes were metabolic pathways and starch and sucrose metabolism (Figure 6D). Particularly, the SMPDL3B correlated genes were also enriched in acute myeloid leukemia, hematopoietic cell lineage, and leukocyte *trans*-endothelial migration (Figure 6D). Moreover, leukemia stem cells play prominent roles in leukemogenesis and propagation due to their capacities of self-renewal, differentiation, and proliferation. Thus, the established markers of leukemia stem cells such as CD123 and CD96 were determined. Spearman’s rank tests showed that the mRNA expression of SMPDL3B was positively correlated with the mRNA expression of CD123, CD96, and CD25 in 151 AML patients, indicating that SMPDL3B may regulate myeloid leukemia development via promoting self-renewal of leukemia stem cells (Figures 7A–C).

## Discussion

AML, characterized by the low cure rate and high relapse, is the most severe adult acute leukemia. Therefore, exploring novel biomarkers and potential therapeutic targets to improve the diagnosis and therapy for human AML was urgently needed. Herein, we suggested for the first time the prognostic and biological significance of the phosphodiesterase SMPDL3B in AML patients. The transcription level of SMPDL3B was significantly elevated in AML patients compared to healthy control or lymphoid leukemia samples. This finding agreed with the results reported in other human cancers including prostate cancer (Waldbillig et al., 2020) and hepatocellular carcinoma (Liu et al., 2020). Curiously, the overexpression of SMPDL3B was associated with some clinicopathological characteristics, including cytogenetics risk (*p* = 0.0014) and karyotypes (*p* < 0.0001). Interestingly, cox multivariate analysis results suggested that SMPDL3B was an independent prognostic factor for the overall survival of AML. Accordantly, Frank W. et al. demonstrated that high expression of SMPLD3B was inversely associated with localized prostate cancer prognosis (Waldbillig et al., 2020). Notably, blocked expression of SMPDL3B significantly inhibited the growth of AML cells *in vivo*. These results suggest that SMPDL3B may be a good prognostic indicator and therapeutic target in human cancers. Further investigations are needed to uncover the prognostic significance of SMPDL3B in other types of cancers.

After the discovery of SMPDL3B and identification of it as a glycosylphosphatidylinositol- (GPI-) anchored protein (Masuishi et al., 2013), several seminal findings dominate thinking about the biological function of SMPDL3B. These findings include the following SMPDL3B modulates podocyte injury phenotypes in glomerular disease by shifting suPAR-mediated podocyte injury from a migratory phenotype to an apoptotic phenotype (Yoo et al., 2015); SMPDL3B functions at the interface of membrane biology and innate immunity via negative regulating Toll-like receptor signaling (Heinz et al., 2015); SMPDL3B impairs insulin signaling by interfering with insulin receptor isoforms binding to caveolin-1 in the plasma membrane (Mallela et al., 2019; Mitrofanova et al., 2019); SMPDL3B is an off-target biomarker of rituximab in focal segmental glomerulosclerosis (Fornoni et al., 2011); and the crystal structure revealed that the active site of SMPDL3B was located in a narrow boot-shaped cavity (Gorelik et al., 2016). Recently, it was reported that SMPDL3B promoted HCC cell growth, invasion, and migration via inducing ceramide hydrolysis and ceramide-1-phosphate production (Liu et al., 2020). Moreover, high expression of SMPLD3B is inversely associated with prognosis in localized prostate Cancer (Waldbillig et al., 2020). These studies provide clues as to the potential function of SMPDL3B, however, both the biological function of SMPDL3B in malignant tumors and its main substrates remain largely unclear. In this study, we firstly found that expression of SMPDL3B was significantly upregulated in human AML samples. In addition, blocked SMPDL3B expression inhibited AML cells growth both *in vitro* and *in vivo* via promoting cell apoptosis. For further interpretation of the mechanism of SMPDL3B contributing to apoptosis of human AML cells, SMPDL3B correlated genes were identified. According to the KEGG pathway analysis, the SMPDL3B correlated genes may affect AML cell apoptosis by regulating starch and sucrose metabolism. Consistently, a previous study showed that SMPDL3B modulates insulin receptor signaling and thereby contributes to the production of ceramide-1-phosphate (Fornoni et al., 2014; Mallela et al., 2019; Mitrofanova et al., 2019). Moreover, macrophage-derived thrombospondin 1 promotes obesity-associated non-alcoholic fatty liver disease through suppressing the expression of SMPDL3B (Gwag et al., 2021). Together, these results indicated that SMPDL3B may regulate starch and sucrose metabolism via modulating insulin signaling in AML cells. Interestingly, SMPDL3B blocks the Toll-like receptor signaling pathway and negatively regulates innate immunity (Heinz et al., 2015), which may partially explain that SMPDL3B correlated genes enriched to IL-17 signaling pathway and AML. Taken together, our results reveal that SMPDL3B promotes that the survival of AML cells may be via regulating glucose metabolism or innate immunity. Further detection of the detailed mechanism of SMPDL3B support development of AML is needed.

However, several limitations to the function and mechanism of SMPDL3B in AML should be noted. Firstly, the prognostic indicator effect of SMPDL3B in AML patients was only examined by using the TCGA data. Future research that enrolls larger AML patients is necessary to further explore the association between SMPDL3B expression and overall survival or disease-free survival in AML patients. Secondly, the mechanism of SMPDL3B supporting AML growth is still elusive. In this study, the mechanism of SMPDL3B function in AML was detected by GO enrichment and KEGG pathway analysis. Although these results are consistent with previous reports, elaborate experimental designs, such as RNA sequencing or protein interaction analysis, are necessary to further explore how SMPDL3B regulates AML development.

Together, in the present study, we showed for the first time that high expression of SMPDL3B was significantly associated with unfavorable outcomes in human AML. Moreover, SMPDL3B might be identified as an independent prognostic biomarker for AML. In addition, deficiency of SMPDL3B significantly inhibited the growth of AML both *in vitro* and *in vivo*. These results indicated that SMPDL3B could serve as a promising indicator and potential therapeutic target for AML patients. Collectively, these findings call for further laboratory experiments and clinical trials to validate SMPDL3B in cancer progression.

## Data Availability

The datasets presented in this study can be found in online repositories. The names of the repository/repositories and accession number(s) can be found in the article/Supplementary Material.

## Abbreviations

AML: acute myeloid leukemia; SMPDL3B: Sphingomyelin Phosphodiesterase Acid Like 3B; GEPIA: Gene Expression Profiling Interactive Analysis; GO: gene ontology; TUNEL: TdT-mediated dUTP Nick-End Labeling; KEGG: Kyoto Encyclopedia of Genes and Genomes; BP: biological processes; CC: cell component; MF: molecular function.
